# Elderly kidney transplant recipients: efficacy and risks of a conservative immunosuppression regimen

**DOI:** 10.1590/2175-8239-JBN-2025-0068en

**Published:** 2026-02-13

**Authors:** Aline Cunha Lima Alcântara, Raoni de Oliveira Domingues-da-Silva, Janaína de Almeida Mota Ramalho, Rafael Ximenes Oliveira, Isvi Brandão Araújo, Sônia Leite da Silva, Claudia Maria Costa de Oliveira, Paula Frassinetti Castelo Branco Camurça Fernandes

**Affiliations:** 1 Universidade Federal do Ceará, Faculdade de Medicina, Hospital Universitário Walter Cantídio, Fortaleza, CE, Brazil.; 2 Universidade de Fortaleza, Faculdade de Medicina, Fortaleza, Ceará, Brazil.; 3 Universidade Estadual do Ceará, Faculdade de Medicina, Fortaleza, Ceará, Brazil.; 4 Universidade Estadual do Ceará, Fortaleza, Ceará, Brazil.

**Keywords:** Immunosuppression, Kidney Transplantation, Elderly

## Abstract

**Introduction:**

Over the past decade, there has been an increase in renal transplantation among the elderly. However, age-related declines in immune function heighten the risks associated with immunosuppressive therapy, impacting patient survival. This study examines the first year’s immunosuppressive regimen post-kidney transplantation in elderly individuals.

**Methodology:**

A singlecenter retrospective cross-sectional study was conducted, categorizing kidney transplant recipients from January 2013 to December 2017 into two groups: a control group (aged 30–50 years) and a seniors group (over 60 years).

**Results:**

The study included 59 seniors and 114 controls. Seniorsreceived lower mycophenolate doses (11 ± 7.7 mg/kg/day) compared to controls (15 ± 7.2 mg/kg/day; p = 0.003) and fewer corticosteroids were administered (64.9% vs. 18.6%; p = 0.0001) as maintenance immunosuppression. Seniors also had a higher tacrolimus level-to-dose ratio (p < 0.05). Additionally, seniors experienced more leukopenia (56.5% vs. 35.6%; p = 0.017) and cytomegalovirus infections (44.7% vs. 26.9%; p = 0.034).

**Conclusion:**

Despite receiving lower mycophenolate doses and fewer corticosteroids along with a higher tacrolimus level-to-dose ratio, seniors had more adverse events. However, graft survival was not affected. These results suggest that elderly patients may benefit from reduced immunosuppressant exposure, supporting a conservative approach.

## Introduction

The prevalence of elderly individuals requiring hemodialysis has tripled over the past two decades, resulting in a corresponding increase in the number of kidney transplant recipients (KTRs) within this age group^1^. Despite that, only half of the elderly patients on the transplant waiting list undergo transplantation due to mortality prior to transplantation or lack of suitable donors^2^.

While transplantation improves survival in older patients, outcomes remain less favorable than in younger recipients^3^. This disparity is partly due to the increased susceptibility of elderly patients to the side effects of immunosuppressive medications used in kidney transplantation^4,5^.

Age-related pharmacologic changes can alter immunosuppressive drug levels and increase the risk of adverse effects in elderly patients^5^. Although immunosenescence may reduce the risk of rejection, it is associated with decreased graft longevity and increased susceptibility to infections and malignancies^6^. These factors highlight the need to tailor immunosuppressive protocols to the needs of these expanding elderly population and to implement individualized monitoring strategies.

Advanced age is often considered an exclusion criterion in clinical trials^7^, and clinical trials involving immunosuppressants fail to reflect the reality of the increasing number of elderly transplant recipients. Therefore, it can be challenging to assess the suitability of these medications for this specific age group.

This study aimed to compare immunosuppressive dosing in elderly versus non-elderly recipients and to evaluate differences in adverse events and clinical outcomes during the first year post-transplant.

## Methods

This was a retrospective study conducted at the Hospital Universitário Walter Cantídio, Ceará, Brazil. Individuals who received a deceased donor kidney transplantation from January 2013 to December 2017 were included in this analysis. Patients aged ≥ 60 years (elderly group) at the time of kidney transplantation were compared to those aged 30 to 50 years (control group). The study protocol was approved by the Ethics and Research Committee (number: 3.250.003). All methods adhered to relevant guidelines and regulations.

Standard immunosuppression at our institution consisted of induction with thymoglobulin (ATG) or basiliximab, followed by a maintenance regimen with tacrolimus (TAC) and mycophenolate or mammalian target of rapamycin (mTOR) inhibitors. All patients received corticosteroids, except for those with zero-HLA mismatches, panel reactive antibodies (PRA) < 30%, children, diabetics, obesity with a body mass index (BMI) > 30 kg/m^2^, liver diseases caused by hepatitis B or C viruses, peripheral arterial disease, severe bone disease, and dyslipidemia. Post-transplant prophylaxis included sulfamethoxazole-trimethoprim for all patients and universal primary prophylaxis for cytomegalovirus (CMV) with ganciclovir or valganciclovir for patients receiving ATG for 100 or 200 days according to CMV serology, except for those with negative donor and recipient serology for CMV.

Our analysis included patients who received a regimen comprising ATG, TAC, and mycophenolate. Individuals who initially received basiliximab (N = 15) or mTOR inhibitors (N = 1), recipients of paired liver-kidney or kidney-pancreas transplants (N = 5), those who used renal perfusion machines (N = 39), those taking medications that affected the level of TAC prior to transplantation (N = 6), and those with leukopenia or thrombocytopenia prior to transplantation and patients with incomplete or insufficient data in their medical chart (N = 19) were excluded. Data were collected from medical records.

In our institution, clinical data for each patient were recorded in an individual follow-up sheet, in which laboratory tests, prescribed medications, and adverse events observed during the follow-up period were systematically documented. The information collected in these records formed the basis for our analysis. We compared immunosuppressant doses, metabolic, infectious, hematologic, allograft rejection rates, glomerular filtration rate (GFR) and both patient and allograft survival between the elderly and the control groups.

Immunosuppressive therapy was assessed at baseline and at regular intervals up to 12 months, with dose adjustments guided by clinical judgment and drug levels. CMV infection was defined by the initiation of antiviral therapy, regardless of PCR status. Bacterial or fungal infections were considered present when antimicrobial therapy was initiated and hospitalization was required. New-onset diabetes after transplant was recorded in previously nondiabetic patients who started hypoglycemic therapy post-transplant. Hematologic parameters were evaluated during thymoglobulin use and throughout follow-up at months 1, 3, 6, 9, and 12. Estimated GFR (eGFR) was calculated using the Modification of Diet in Renal Disease (MDRD) equation.

This study was developed as part of the Professional Master’s Program in Kidney Transplantation at the State University of Ceará.

### Statistical Analysis

Continuous variables were summarized using medians and interquartile ranges (IQRs) or means and standard deviations (SDs) as appropriate, whereas categorical variables were summarized as frequencies and percentages. Student’s t-test, Mann-Whitney test, or Fisher’s test were used to compare elderly and control groups as appropriate. Values of p < 0.05 were considered statistically significant.

To analyze the eGFR, we used a multi-step approach to handle missing data. This involved assigning an eGFR value of zero mL/min/1.73 m^2^ to those who died with non-functioning grafts or had nephrectomies before the 12th month. For death with a functioning graft, the last observation carried forward (LOCF) method was used. Any remaining missing eGFR value was handled using multiple imputation by chained equations (MICE).

A multivariate analysis was conducted to explore factors associated with mortality. Age, as a continuous variable, and variables that demonstrated statistically significant differences (p < 0.05) between the elderly and young groups were selected for inclusion. In multivariate analysis, Cox regression was employed to identify independent predictors of mortality. Variables were entered into the model using a backward stepwise method. Multicollinearity was assessed through variance inflation factors (VIFs), and interactions between significant variables were examined.

For assessing graft and patient survival, a competing risk analysis using the Fine-Gray model was employed.

Data analysis was performed using the Statistical Package for the Social Sciences (SPSS) version 22 for Mac.

## Results

A total of 272 patients within the specified age groups received kidney transplants at our center from January 2013 to December 2017. Thirteen subjects had insufficient data in their medical records, and a total of 86 subjects met one or more exclusion criteria. A total of 173 KTRs were included in our analysis, 59 in the elderly group and 114 in the control group.

The demographic data, comorbidities, immunological, and donor data according to the age group are shown in Table 1. The main causes of underlying kidney disease in the control group were chronic glomerulonephritis, systemic arterial hypertension (SAH), and unknown causes. In contrast, the main causes in the elderly group were diabetes mellitus (DM) and SAH. The prevalence of DM and cardiovascular diseases was higher in the elderly group.

**Table 1:** Baseline demographic and clinical characteristics of the total cohort, control group, and elderly group

Variables		Total (N = 173)	Control (N = 114)	Elderly (N = 59)	p
Receptor					
Age (years)		49.1 ± 13.4	**40.5 ± 6.7**	**65.8 ± 4.2**	**< 0.05**
Male (n, %)		113 (65)	73 (64)	40 (67.7)	> 0.05
BMI (kg/m^2^)		25.1 ± 4.3	24.6 ± 4.5	26.2 ± 3.6	> 0.05
Cause of kidney disease (n, %)	GN	34 (19.6)	**33 (29)**	**1 (1.7)**
	DM	36 (20.8)	**8 (7)**	**28 (47.5)**
	SAH	33 (19)	20 (17.5)	13 (22)	**0,0001**
	PKD	15 (8.6)	7 (6.1)	8 (13.5)	
	Others	11 (6.3)	8 (7)	3 (5)
	IND	44 (25)	**38 (33.3)**	**6 (10.2)**
Comorbidities	>1	70 (40.4)	**35 (30.7)**	**35 (59.3)**	**0.0001**
Diabetes Mellitus	DM	36 (20.8)	**8 (7)**	**28 (47.5)**	**0.0001**
Cardiovascular disease (n, %)		36 (20.8)	**16 (14)**	**20 (33.8)**	**< 0.05**
TDBTx (months)		42.7	44.2 ± 45.1	41.3 ± 26.9	> 0.05
Previous blood transfusion (n, %)		104 (60.1)	74 (65)	30 (50.8)	> 0.05
Retransplantation (n, %)		7 (4)	6 (5.3)	1 (1.7)	> 0.05
HLA mismatches (n, %)	1	5 (2.8)	2 (1.8)	3 (5.1)	
	2	22 (12.7)	**22 (19.3)**	**0 (0.0)**	
	3	53 (30.6)	36 (31.6)	17 (28.8)	**0,001**
	4	54 (31.2)	36 (31.6)	18 (30.5)		
	5	29 (16.7)	**12 (10.5)**	**17 (28.8)**	
	6	10 (5.7)	6 (5.3)	4 (6.8)	
PRA high risk >20%	Class I	29 (16.7)	**23 (20.2)**	**6 (10.2)**	**0.095**
	Class II	17 (9.8)	10 (8.8)	7 (11.9)	> 0.05
DSA > 500 (n, %)		32 (18.4)	**27 (23.7)**	**5 (8.5)**	**0.015**
**Donor**				
Age (years)		32.7 ± 12.6	31.9 ± 12.3	34.2 ± 13.4	> 0.05
Male (n, %)		126 (72.8)	84 (73.7)	42 (71.2)	> 0.05
ECD (n, %)		2 (1,1)	0	2 (3,4)
Final Cr (mg/dL)		1.27 ± 0.7	1.28 ± 0.74	1.25 ± 0.62	> 0.05
Cause of death (n, %)	Trauma	125 (72.2)	86 (75.4)	39 (66.1)	> 0.05
	Cerebrovascular	38 (21.9)	20 (17.5)	18 (30.5)
	Other	10 (5.7)	8 (7.0)	2 (3.4)
Kidney Bx MAPI	0	33 (19)	22 (19.2)	11 (18.6)	> 0,05
	1 –7	11 (6.3)	7 (6.1)	4 (6.7)	

Abbreviations – BMI: body mass index; GN: glomerulomnephritis; DM: diabetes mellitus; SAH: systemic hypertension; PKD: polycystic kidney disease; IND: indeterminate; TDBT: time on dialysis before transplant; HLA: human leukocyte antigen; PRA: panel reactive antibody; DSA: donor specific antibody; ECD: expanded criteria donor; Cr: creatinine; MAPI: Maryland aggregate pathology index. Note – continuous variables presented as means ± standard deviations.

Fewer patients in the elderly group had donor-specific antibody (DSA) titers with mean fluorescence greater than 500. The elderly group received renal grafts with a higher number of human leukocyte antigens (HLA) mismatches compared to the control group.

Donor characteristics were similar between groups (Table 1). In this study, only two donors met the criteria for expanded criteria donors, and none were aged 60 years or older.

Analysis of transplantation-related variables revealed that the length of hospital stay (LHS) was significantly longer in the elderly group, while the cold ischemia time (CIT), the incidence of delayed graft function (DGF), and serum creatinine at hospital discharge were similar between the two groups (Table 2).

**Table 2:** Analysis of transplantation-related variables in the total cohort, control group, and elderly group

Variables	Total (N = 173)	Control (N = 114)	Elderly (N = 59)	p
CIT (hours, mean ± SD)	21.3 ± 4	22 ± 4	21 ± 4	> 0.05
DGF (n, %)	112 (64.7)	74 (64.9)	38 (64.4)	> 0.05
LHS (days, mean ± SD)	19.2 ± 13,4	17.8 ± 11.4	22 ± 16.3	0.045
THD (days, median)	7 (1 –60)	7 (1 –60)	10 (1 –47)	–
Crh (mg/dL, mean ± SD)	2.25 ± 1.42	2.25 ± 1.3	2.26 ± 1.66	> 0.05

Abbreviations – CIT: cold ischemia time; DGF: delayed graft function; LHS: length of hospital stay; THD: time on dialysis; Crh: creatinine at hospital discharge.

### Differences in Immunosuppressive Regimen

The total dose of ATG (approximately 5.5 mg/kg) did not differ between the groups. Corticosteroid use in the maintenance regimen was less common in the elderly group (elderly: 18.6% vs. control: 64.9%; p = 0.0001).

The rate of modifications or discontinuation of maintenance immunosuppression was similar between groups (27.1% in elderly vs. 16.6% in controls, p > 0.05), with CMV infection being the leading cause for regimen changes.

Mycophenolate doses after the third month were significantly lower in the elderly group compared to controls. At months 3, 6, and 12, elderly recipients received 11.6 ± 8.1, 10.8 ± 8.5, and 10.9 ± 8.7 mg/kg/day, respectively, versus 15.8 ± 7.0, 14.7 ± 7.6, and 14.9 ± 8.0 mg/kg/day in the control group (p = 0.001, 0.006, and 0.008, respectively). Mean tacrolimus doses were significantly lower in the elderly group compared to controls at 3, 6, and 12 months: 0.050 ± 0.031, 0.039 ± 0.025, and 0.034 ± 0.021 mg/kg/day vs. 0.068 ± 0.038, 0.060 ± 0.034, and 0.053 ± 0.031 mg/kg/day, respectively (p = 0.004, 0.0001, 0.001). Despite the lower doses, tacrolimus trough levels were similar between groups at all time points, except at month 6, when levels were significantly higher in the elderly group (7.3 ± 4.2 vs. 5.9 ± 1.8 ng/mL; p < 0.05).

### Adverse Events after Transplantation

In the first month post-transplant, fungal or bacterial infection rates did not differ significantly between the groups (44% in elderly and 47.3% in controls, p > 0.05). Among these infections, urinary tract infections were the most frequently observed (51.2%). Estimated GFR at the end of the first month did not differ between patients who did and did not experience a urinary tract infection during that period (39 [29–61] vs. 46 [26–60] mL/min/1.73 m^2^; p = 0.7).

Table 3 outlines the main adverse events observed after the first month of transplantation. Leukopenia was found to be more prevalent in the elderly group compared to the control group (56.5% vs 35.6%; p = 0.017). Lymphopenia persisted at the end of the first year in 27.9% of the elderly group and 14.6% of the control group (p > 0.05). The prevalence of thrombocytopenia, on the other hand, was similar between groups (19%).

**Table 3:** Post-transplant complications in the total cohort, control group, and elderly group

Variables	Total (n, %)	Control (n, 95% CI)	Elderly (n, 95% CI)	p
Leukopenia	62 (42.1)	36 (35.6, 26.9 –4.2)	26 (56.5, 43.7 –69.3)	0.017
Thrombocytopenia	28 (19)	19 (18.8, 11.7 –25.9)	9 (19.5, 9.3 –29.7)	> 0.05
Nephrotoxicity	6 (4)	4 (3.8, 0.0 –7.8)	2 (4.2, 0.0 –9.9)	> 0.05
NODAT	18 (13.1)	13 (12.2, 6.2 –18.2)	5 (16.1, 5.9 –26.3)	> 0.05
CMV	49 (32.4)	28 (26.9, 18.7 –35.1)	21 (44.7, 31.8 –57.6)	0.034
Bacterial/fungal infection	43 (28.6)	25 (24.0, 16.0 –32.0)	18 (39.1, 26.4 –51.8)	0.059
Neoplasia	3 (2)	1 (1.0, 0.0 –2.9)	2 (4.2, 0.0 –9.9)	> 0.05
Others	07 (4.6)	5 (4.8, 1.0 –8.6)	2 (4.2, 0.0 –9.9)	> 0.05

Abbreviations – NODAT: new-onset diabetes after transplant; CMV: cytomegalovirus.

The frequency of NODAT was 16.1% in the elderly group and 12.2% in the control group (p > 0.05). CMV infection was more common in the elderly group (44.7% vs. 26.9%; p = 0.034), and there was a tendency towards a higher occurrence of bacterial and/or fungal infections requiring hospitalization in this group (39.1% vs. 24%; p = 0.059).

### Post-Transplant Outcomes

**Figure 1:**
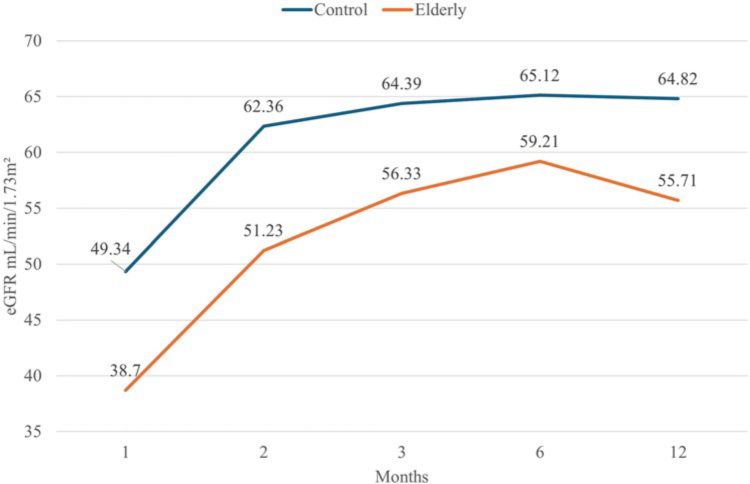
Estimated GFR in the 12 months after transplantation.

Table 4 summarizes post-transplant outcomes. Treated acute rejection occurred in 3.7% of elderly patients and 3.6% of controls (p > 0.05). Graft nephrectomy was performed in 10% of both groups. No difference between groups was found in the 12-month eGFR (57.7 ± 29.6 vs. 66.2 ± 29.1 mL/min/1.73 m^2^, p = 0.075). Figure 1 depicts the eGFR among the living patients during the 12-month follow-up.

**Table 4:** Outcomes summarizing post-transplant results in the total cohort, control group, and elderly group

Variables	Total (n, %)	Control (n, 95% CI)	Elderly (n, 95% CI)	p
Acute Rejection	6 (3.7)	4 (3.6, 0.1 –7.1)	2 (3.7, 0.0 –9.1)	> 0.05
Graft Nephrectomy	10 (6.1)	7 (6.4, 1.8 –11.0)	3 (5.6, 0.0 –11.7)	
Vascular	5 (3)	3 (2.7, 0.0 –5.8)	2 (3.7, 0.0 –9.1)	
Non-primary function	2 (1.2)	2 (1.8, 0.0 –4.4)	0 (0)	> 0.05
Urinary fistula	1 (0.6)	1 (0.9, 0.0 –2.8)	0 (0)	
Hemorrhagic shock	1 (0.6)	1 (0.9, 0.0 –2.8)	0 (0)	
Indeterminate	1 (0.6)	0 (0)	1 (1.8, 0.0 –5.3)	
Death	13 (8)	5 (4.6, 0.8 –8.4)	8 (15.1, 5.8 –24.4)	
Cardiovascular	5 (3)	1 (0.9, 0.0 –2.8)	4 (7.5, 0.7 –14.3)	
Infection	4 (2.4)	2 (1.8, 0.0 –4.4)	2 (3.7, 0.0 –9.1)	**0.021**
Hemorrhagic shock	3 (1.8)	2 (1.8, 0.0 –4.4)	1 (1.8, 0.0 –5.3)	
Indeterminate	1 (0.6)	0 (0)	1 (1.8, 0.0 –5.3)	

**Figure 2:**
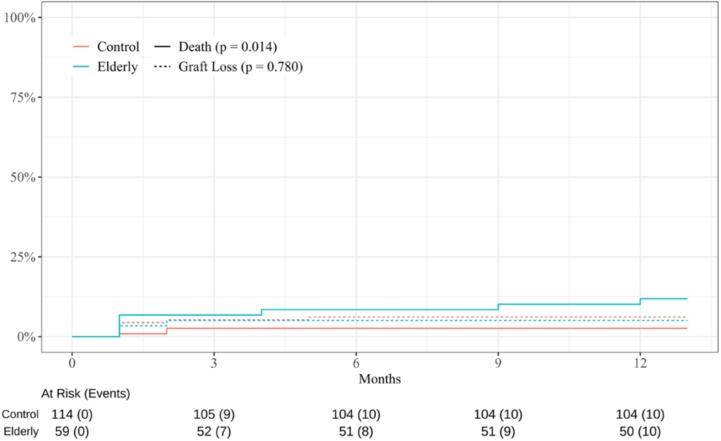
Competing risk analysis between patient death and graft loss.

In the elderly cohort, five patients died within the first week post-transplant, compared to only two cases of early mortality in the control group. At the end of the first year, the elderly group had an increased risk of dying (sHR: 4.65, 95% CI: 1.22 – 17.7, p = 0.024) and exhibited lower overall survival compared to the control group (86.4% vs 95.6%; p = 0.014). However, graft survival did not differ significantly between the groups (elderly: 86.8% vs control: 92.7%; p = 0.780) (Figure 2). In the multivariate analysis, including age, diabetes, panel reactive antibody > 20%, body mass index, and prior cardiovascular disease, no variable was independently associated with mortality.

## Discussion

In our analysis, we found that elderly KTRs were more likely to receive a corticosteroid-free maintenance immunosuppressive regimen and were administered significantly lower doses of mycophenolate compared to their younger counterparts. Although they had similar tacrolimus blood levels, higher tacrolimus level-to-dose ratios were observed in the elderly. While their death-censored one-year graft survival was similar to that of the controls, they experienced a lower one-year overall survival rate driven mainly by deaths in the first-week post-transplantation.

Our institution’s immunosuppression protocol does not explicitly exclude elderly patients from corticosteroid regimens. However, prednisone was used less frequently in our elderly patients, likely due to a higher prevalence of diabetes and a lower immunological risk. In contrast, the control group had a greater incidence of DSA before transplantation, aligning with the observation that younger patients typically face a higher immunological risk^8^. Calcineurin inhibitors may allow corticosteroid avoidance or early corticosteroids withdrawal without increasing rejection risk in low-immunologic-risk patients. Given the elderly’s heightened vulnerability to NODAT, cardiovascular disease, and osteoporosis, this approach is especially beneficial in this population^9,10^. It may improve long-term patient survival and help mitigate these comorbidities^11^. In fact, despite their higher baseline risk, our elderly patients had an incidence of NODAT similar to that of the controls.

Although our protocol does not adjust mycophenolate doses based on age, lower doses in the elderly likely reflected higher rates of leukopenia, CMV infection, and increased bacterial/fungal infections requiring hospitalization after the first post-transplant month. A lower mean dose of mycophenolate mofetil in the elderly at 1-year posttransplantation has been reported before^12^, although in that study CMV infection rates had been similar in the elderly and non-elderly group. Importantly, reduced mycophenolate doses and steroid use in our elderly cohort were not associated with a significant increase in rejection rates. Moreover, in a previous study, lower exposure to immunosuppressive medications was associated with better graft survival in elderly individuals, even after accounting for deaths^13^. Conversely, higher exposure was linked to an increased risk of death and graft loss.

After the third month of transplantation, the elderly cohort showed a pronounced difference in the level-to-dose ratio of TAC compared to the control group. Jacobson et al.^14^ observed a 68% lower TAC level-to-dose ratio in the elderly compared to younger individuals (129.8 vs 77.1 ng/mL/mg/kg; p < 0.05). Likewise, David-Neto et al.^15^ demonstrated that the average TAC dose was significantly lower in elderly individuals (8.6 ± 4.8 mg vs. 12.1 ± 5.1 mg; p < 0.05) to achieve blood TAC levels comparable to those in younger patients.

In our cohort, treated acute rejection rates were comparable despite the elderly group having more HLA mismatches, likely due to allocation policies favoring donors over 50 for older recipients, a common practice among transplant centers. A rejection rate of 10% in patients over 65 years old, which is half the rate observed in those between 20 and 30 years old, has been reported by Tullius et al.^6^. The low rejection rate (3.6% in both groups) compared to published data may reflect high immunosuppressant exposure, fewer high-risk recipients and donors, and the study’s limited sample size.

The prevalence of CMV infection was significantly higher in the elderly group compared to the control group (44.7 vs. 26.9%). Research indicates that CMV infection occurs in over 60% of high-risk transplant patients^16^. Mycophenolate has been shown to increase the risk of CMV infection in the elderly population^13^, whereas mTOR inhibitors appear to offer a protective effect against CMV^17^. In older adults, mTOR inhibitors demonstrate stable pharmacokinetics during the first six months post-transplant, reaching steady drug levels by day seven, indicating that major dose adjustments are generally unnecessary^15^.

International data have not consistently shown significant differences in overall patient and graft survival rates at 1- and 5-years post-transplantation^12^. However, the lower survival in our elderly group is consistent with findings from a previous Brazilian study that reported similar survival differences between elderly and younger recipients^18^. This disparity has been attributed to the increased burden of comorbidities such as diabetes, which is a well-recognized risk factor for premature mortality following kidney transplantation^19^. Furthermore, the relative risk of death due to infection has been reported to be strongly influenced by age^4^. In our study, most elderly deaths occurred within the first week post-transplant, mainly from cardiovascular causes and infections. Despite higher baseline diabetes and cardiovascular disease, no factor independently predicted mortality, underscoring the complex interplay of variables — potentially including unmeasured factors like frailty — in elderly 12-month post-transplant outcomes. Importantly, previous data have shown that, although elderly KTRs have an increased risk of early post-transplant mortality, they experience a progressive and substantial improvement in long-term survival and are expected to have superior survival rates compared with remaining on dialysis^20^.

While limited by its retrospective design and incomplete data on adherence, immune status, and geriatric frailty, this study benefits from a standardized immunosuppressive protocol and consistent donor profiles.

Our findings suggest that elderly KTRs might benefit from a reduced exposure to immunosuppressive agents, aligning with existing literature. Careful candidate selection and tailored perioperative care remain essential to optimizing outcomes in this growing patient population.

## Data Availability

The full dataset supporting the findings of this study is available upon request from the corresponding author, Aline Cunha Lima Alcântara. The dataset is not publicly available due to the presence of sensitive clinical information that could compromise participant privacy.
