# Supplementary material for: Elderly kidney transplant recipients: efficacy and risks of a conservative immunosuppression regimen
Source: J Bras Nefrol. 2026 Feb 13;48(2):e20250068. doi: 10.1590/2175-8239-JBN-2025-0068en (PMC12904329; doi:10.1590/2175-8239-JBN-2025-0068en)
Supplement: Translation

# Translation: Receptores idosos de transplante renal: eficácia e riscos de um regime de imunossupressão conservador

**DOI:** 10.1590/2175-8239-JBN-2025-0068pt

**Authors:** Aline Cunha Lima Alcântara, Raoni de Oliveira Domingues-da-Silva, Janaína de Almeida Mota Ramalho, Rafael Ximenes Oliveira, Isvi Brandão Araújo, Sônia Leite da Silva, Claudia Maria Costa de Oliveira, Paula Frassinetti Castelo Branco Camurça Fernandes

**Affiliations:** 1 Universidade Federal do Ceará, Faculdade de Medicina, Hospital Universitário Walter Cantídio, Fortaleza, CE, Brasil.; 2 Universidade de Fortaleza, Faculdade de Medicina, Fortaleza, Ceará, Brasil.; 3 Universidade Estadual do Ceará, Faculdade de Medicina, Fortaleza, Ceará, Brasil.; 4 Universidade Estadual do Ceará, Fortaleza, Ceará, Brasil.

**Keywords:** Imunossupressão, Transplante Renal, Idosos

## Abstract

**Introdução:**

Ao longo da última década, verificou-se um aumento no número de transplantes renais entre idosos. No entanto, o declínio da função imunológica associado ao envelhecimento eleva os riscos associados à terapia imunossupressora, com impacto na sobrevida dos pacientes. Este estudo analisa o regime imunossupressor adotado no primeiro ano após o transplante renal em indivíduos idosos.

**Metodologia:**

Foi realizado um estudo transversal retrospectivo, de centro único, categorizando receptores de transplante renal de janeiro de 2013 a dezembro de 2017 em dois grupos: um grupo controle (com idades entre 30 e 50 anos) e um grupo de idosos (com idade superior a 60 anos).

**Resultados:**

O estudo incluiu 59 idosos e 114 controles. Os idosos receberam doses mais baixas de micofenolato (11 ± 7,7 mg/kg/dia) em comparação aos controles (15 ± 7,2 mg/kg/dia; p = 0,003) e menores quantidades de corticosteroides foram administradas (64,9% vs. 18,6%; p = 0,0001) como imunossupressão de manutenção. Essa população também apresentou uma relação nível/dose de tacrolimus mais elevada (p < 0,05). Além disso, os pacientes idosos apresentaram mais casos de leucopenia (56,5% vs. 35,6%; p = 0,017) e infecções por citomegalovírus (44,7% vs. 26,9%; p = 0,034).

**Conclusão:**

Apesar de receberem doses mais baixas de micofenolato e menos corticosteroides, juntamente com uma relação nível/dose de tacrolimus mais elevada, os idosos apresentaram maior ocorrência de eventos adversos. Entretanto, a sobrevida do enxerto não foi afetada. Esses resultados sugerem que pacientes idosos podem se beneficiar de uma exposição reduzida a imunossupressores, o que reforça uma abordagem conservadora.

## Introdução

A prevalência de indivíduos idosos que necessitam de hemodiálise triplicou nas últimas duas décadas, resultando em um aumento correspondente no número de receptores de transplante renal (RTR) nessa faixa etária^1^. Apesar disso, somente cerca de metade dos pacientes idosos na lista de espera para transplante são submetidos ao procedimento devido à mortalidade pré-transplante ou à falta de doadores compatíveis^2^.

Embora o transplante melhore a sobrevida em pacientes de idade mais avançada, os desfechos continuam sendo menos favoráveis do que em receptores mais jovens^3^. Essa disparidade se deve, em parte, à maior suscetibilidade dos pacientes idosos aos efeitos adversos dos medicamentos imunossupressores que são utilizados no transplante renal^4,5^.

Alterações farmacológicas relacionadas à idade podem alterar os níveis de medicamentos imunossupressores e aumentar o risco de efeitos adversos em pacientes idosos^5^. Embora a imunossenescência possa reduzir o risco de rejeição, ela está associada à redução da longevidade do enxerto e ao aumento da suscetibilidade a infecções e neoplasias^6^. Esses fatores destacam a necessidade de adequar os protocolos imunossupressores às necessidades dessa população idosa, que se encontra em expansão, bem como implementar estratégias de monitoramento individualizadas.

A idade avançada é frequentemente considerada um critério de exclusão em ensaios clínicos^7^, e os ensaios que envolvem imunossupressores não refletem a realidade do número crescente de receptores de transplantes idosos. Portanto, pode ser desafiador avaliar a adequação desses medicamentos para essa faixa etária específica.

Este estudo teve como objetivo comparar a dosagem imunossupressora em receptores idosos e não idosos e avaliar as diferenças nos eventos adversos e desfechos clínicos durante o primeiro ano pós-transplante.

## Métodos

Este foi um estudo retrospectivo realizado no Hospital Universitário Walter Cantídio, Ceará, Brasil. Foram incluídos nesta análise os indivíduos que receberam transplante renal de doador falecido no período de janeiro de 2013 a dezembro de 2017. Pacientes com idade ≥ 60 anos (grupo de idosos) no momento do transplante renal foram comparados àqueles com idade entre 30 e 50 anos (grupo controle). O protocolo do estudo foi aprovado pelo Comitê de Ética e Pesquisa (número: 3.250.003). Todos os métodos seguiram as diretrizes e normas relevantes.

O regime imunossupressor padrão em nossa instituição consistiu na indução com timoglobulina (ATG) ou basiliximabe, seguida por terapia de manutenção com tacrolimus (TAC) e micofenolato ou inibidores da enzima alvo da rapamicina em mamíferos (mTOR). Todos os pacientes receberam corticosteroides, exceto aqueles com zero incompatibilidade HLA, painel de reatividade de anticorpos (PRA) < 30%, crianças, diabéticos, obesidade com índice de massa corporal (IMC) > 30 kg/m^2^, doenças hepáticas causadas pelos vírus da hepatite B ou C, doença arterial periférica, doença óssea grave e dislipidemia. A profilaxia pós-transplante incluiu sulfametoxazol-trimetoprima para todos os pacientes e profilaxia primária universal para citomegalovírus (CMV) com ganciclovir ou valganciclovir para pacientes que receberam ATG por 100 ou 200 dias, de acordo com a sorologia para CMV, com exceção daqueles com sorologia negativa tanto do doador quanto do receptor.

Nossa análise incluiu pacientes que receberam um regime composto por ATG, TAC e micofenolato. Foram excluídos indivíduos que inicialmente receberam basiliximabe (N = 15) ou inibidores de mTOR (N = 1), receptores de transplante combinado fígado-rim ou rim-pâncreas (N = 5), aqueles que utilizaram máquinas de perfusão renal (N = 39), pacientes em uso de medicamentos que influenciaram o nível de TAC antes do transplante (N = 6), bem como aqueles com leucopenia ou trombocitopenia pré-transplante e pacientes com dados incompletos ou insuficientes em prontuário (N = 19). As informações foram coletados a partir dos prontuários médicos.

Em nossa instituição, os dados clínicos de cada paciente foram registrados em uma ficha individual de acompanhamento, na qual os exames laboratoriais, os medicamentos prescritos e os eventos adversos observados durante o período de acompanhamento foram sistematicamente documentados. As informações coletadas nesses registros constituíram a base para nossa análise. Foram comparadas as doses de imunossupressores, taxas metabólicas, infecciosas, hematológicas, de rejeição de aloenxertos, taxa de filtração glomerular (TFG) e sobrevida do paciente e do aloenxerto entre os grupos de idosos e controle.

A terapia imunossupressora foi avaliada no período basal e em intervalos regulares até 12 meses, com ajustes de dose orientados pelo julgamento clínico e pelos níveis do medicamento. A infecção por CMV foi definida pelo início de terapia antiviral, independentemente do status da PCR. Infecções bacterianas ou fúngicas foram consideradas presentes quando a terapia antimicrobiana foi iniciada e houve necessidade de hospitalização. O diabetes de início recente pós-transplante (NODAT) foi registrado em pacientes anteriormente não diabéticos que iniciaram terapia antidiabética após o procedimento. Os parâmetros hematológicos foram avaliados durante o uso de timoglobulina e ao longo do acompanhamento nos meses 1, 3, 6, 9 e 12. A taxa de filtração glomerular estimada (TFGe) foi calculada utilizando a equação MDRD (*Modification of Diet in Renal Disease*).

Este estudo foi desenvolvido no âmbito do Programa de Mestrado Profissional em Transplante Renal da Universidade Estadual do Ceará.

### Análise Estatística

As variáveis contínuas foram resumidas por meio de medianas e intervalos interquartis (IIQ) ou médias e desvios-padrão (DP), enquanto as variáveis categóricas foram descritas como frequências e porcentagens. A comparação entre os grupos de idosos e controle foi realizada por meio dos testes t de Student, Mann–Whitney ou Fisher, de acordo com a natureza das variáveis. Valores de p < 0,05 foram considerados estatisticamente significativos.

Para analisar a TFGe, utilizamos uma abordagem em múltiplas etapas para tratamento de dados ausentes. Isso envolveu atribuir um valor de TFGe de zero mL/min/1,73 m^2^ àqueles que evoluíram a óbito com enxertos não funcionantes ou que foram submetidos à nefrectomia antes do 12º mês. Para óbitos com enxertos funcionantes, utilizou-se o método *Last Observation Carried Forward* (LOCF). Quaisquer valores ausentes de TFGe remanescentes foram tratados por meio de imputação múltipla por equações encadeadas (MICE).

Realizou-se análise multivariada para explorar os fatores associados à mortalidade. Foram selecionadas para inclusão a idade, como variável contínua, e as variáveis que demonstraram diferenças estatisticamente significativas (p < 0,05) entre os grupos de idosos e jovens. Na análise multivariada, empregou-se a regressão de Cox para identificar preditores independentes de mortalidade. As variáveis foram inseridas no modelo utilizando o método *stepwise backward*. A multicolinearidade foi avaliada por meio de fatores de inflação da variância (VIF), e foram examinadas as interações entre variáveis significativas.

Para a avaliação da sobrevida do enxerto e do paciente, foi empregada uma análise de risco competitivo utilizando o modelo *Fine-Gray*.

A análise dos dados foi realizada utilizando o *Statistical Package for the Social Sciences* (SPSS) versão 22 para Mac.

## Resultados

No período de janeiro de 2013 a dezembro de 2017, um total de 272 pacientes dentro das faixas etárias especificadas receberam transplantes renais em nosso centro. Treze indivíduos apresentaram dados insuficientes em seus prontuários médicos e 86 deles preencheram um ou mais critérios de exclusão. Assim, 173 RTR foram incluídos em nossa análise, sendo 59 no grupo de idosos e 114 no grupo controle.

Os dados demográficos, as comorbidades, as características imunológicas e as informações dos doadores de acordo com a faixa etária estão apresentados na Tabela 1. As principais causas de doença renal subjacente no grupo controle foram glomerulonefrite crônica, hipertensão arterial sistêmica (HAS) e causas desconhecidas. Em contrapartida, as principais causas no grupo de idosos foram diabetes mellitus (DM) e HAS. A prevalência de DM e doenças cardiovasculares foi mais elevada no grupo de idosos.

**Tabela 1:** Características demográficas e clínicas basais da coorte total, do grupo controle e do grupo de idosos

Variáveis		Total (N = 173)	Controle (N = 114)	Idosos (N = 59)	p
Receptor					
Idade (anos)		49,1 ± 13,4	**40,5 ± 6,7**	**65,8 ± 4,2**	**< 0,05**
Masculino (n, %)		113 (65)	73 (64)	40 (67,7)	> 0,05
IMC (kg/m^2^)		25,1 ± 4,3	24,6 ± 4,5	26,2 ± 3,6	> 0,05
Causa de doença renal (n, %)	GN	34 (19,6)	**33 (29)**	**1 (1,7)**	
	DM	36 (20,8)	**8 (7)**	**28 (47,5)**	
	HAS	33 (19)	20 (17,5)	13 (22)	**0,0001**
	DRP	15 (8,6)	7 (6,1)	8 (13,5)		
	Outras	11 (6,3)	8 (7)	3 (5)	
	IND	44 (25)	**38 (33,3)**	**6 (10,2)**	
Comorbidades	>1	70 (40,4)	**35 (30,7)**	**35 (59,3)**	**0,0001**
Diabetes Mellitus	DM	36 (20,8)	**8 (7)**	**28 (47,5)**	**0,0001**
Doença cardiovascular (n, %)		36 (20,8)	**16 (14)**	**20 (33,8)**	**< 0,05**
TDBTx (meses)		42,7	44,2 ± 45,1	41,3 ± 26,9	> 0,05
Transfusão sanguínea prévia (n, %)		104 (60,1)	74 (65)	30 (50,8)	> 0,05
Retransplante (n, %)		7 (4)	6 (5,3)	1 (1,7)	> 0,05
Incompatibilidades HLA (n, %)	1	5 (2,8)	2 (1,8)	3 (5,1)	
	2	22 (12,7)	**22 (19,3)**	**0 (0,0)**	
	3	53 (30,6)	36 (31,6)	17 (28,8)	**0,001**
	4	54 (31,2)	36 (31,6)	18 (30,5)		
	5	29 (16,7)	**12 (10,5)**	**17 (28,8)**	
	6	10 (5,7)	6 (5,3)	4 (6,8)	
PRA de alto risco: > 20%	Classe I	29 (16,7)	**23 (20,2)**	**6 (10,2)**	**0,095**
	Classe II	17 (9,8)	10 (8,8)	7 (11,9)	> 0,05
DSA > 500 (n, %)		32 (18,4)	**27 (23,7)**	**5 (8,5)**	**0,015**
Doador					
Idade (anos)		32,7 ± 12,6	31,9 ± 12,3	34,2 ± 13,4	> 0,05
Masculino (n, %)		126 (72,8)	84 (73,7)	42 (71,2)	> 0,05
DCE (n, %)		2 (1,1)	0	2 (3,4)	
Cr final (mg/dL)		1,27 ± 0,7	1,28 ± 0,74	1,25 ± 0,62	> 0,05
Causa do óbito (n, %)	Trauma	125 (72,2)	86 (75,4)	39 (66,1)
	Cerebrovascular	38 (21,9)	20 (17,5)	18 (30,5)	> 0,05
	Outra	10 (5,7)	8 (7,0)	2 (3,4)
Bx renal – MAPI	0	33 (19)	22 (19,2)	11 (18,6)	> 0,05
	1–7	11 (6,3)	7 (6,1)	4 (6,7)	

Abreviaturas – IMC: índice de massa corporal; GN: glomerulonefrite; DM: diabetes mellitus; HAS: hipertensão arterial sistêmica; DRP: doença renal policística; IND: indeterminada; TDBTx: tempo em diálise antes do transplante; HLA: antígeno leucocitário humano; PRA: painel de reatividade de anticorpos; DSA: anticorpo específico contra o doador; DCE: doador com critérios expandidos; Cr: creatinina; MAPI: *Maryland aggregate pathology index*. Nota – variáveis contínuas apresentadas como médias ± desvios-padrão.

Um número menor de pacientes no grupo de idosos apresentou títulos de anticorpos específicos contra o doador (DSA) com média de fluorescência superior a 500. O grupo de idosos recebeu enxertos renais com maior número de incompatibilidades de antígenos leucocitários humanos (HLA) em comparação ao grupo controle.

As características dos doadores foram semelhantes entre os grupos (Tabela 1). Neste estudo, apenas dois doadores atenderam aos critérios para doadores com critérios expandidos, e nenhum apresentava idade igual ou superior a 60 anos.

A análise das variáveis relacionadas ao transplante revelou que o tempo de permanência hospitalar (TPH) foi significativamente mais longo no grupo de idosos, enquanto o tempo de isquemia fria (TIF), a incidência de função retardada do enxerto (FRE) e a creatinina sérica na alta hospitalar foram semelhantes entre os dois grupos (Tabela 2).

**Tabela 2:** Análise das variáveis relacionadas ao transplante na coorte total, no grupo controle e no grupo de idosos

Variáveis	Total (N = 173)	Controle (N = 114)	Idosos (N = 59)	p
TIF (horas, média ± DP)	21,3 ± 4	22 ± 4	21 ± 4	> 0,05
FRE (n, %)	112 (64,7)	74 (64,9)	38 (64,4)	> 0,05
TPH (dias, média ± DP)	19,2 ± 13,4	17,8 ± 11,4	22 ± 16,3	0,045
TD (dias, mediana)	7 (1–60)	7 (1–60)	10 (1–47)	–
Crh (mg/dL, média ± DP)	2,25 ± 1,42	2,25 ± 1,3	2,26 ± 1,66	> 0,05

Abreviaturas – TIF: tempo de isquemia fria; FRE: função retardada do enxerto; TPH: tempo de permanência hospitalar; TD: tempo em diálise; Crh: creatinina na alta hospitalar.

### Difereças no Regime Imunossuppressor

A dose total de ATG (aproximadamente 5,5 mg/kg) não diferiu entre os grupos. O uso de corticosteroides no regime de manutenção foi menos comum no grupo de idosos (idosos: 18,6% vs. controle: 64,9%; p = 0,0001).

A taxa de modificações ou descontinuação da imunossupressão de manutenção foi semelhante entre os grupos (27,1% nos idosos vs. 16,6% nos controles; p > 0,05), sendo a infecção por CMV a principal causa de alterações no regime.

As doses de micofenolato após o terceiro mês foram significativamente mais baixas no grupo de idosos em comparação aos controles. Nos meses 3, 6 e 12, os receptores idosos receberam, respectivamente, 11,6 ± 8,1; 10,8 ± 8,5 e 10,9 ± 8,7 mg/kg/dia, versus 15,8 ± 7,0; 14,7 ± 7,6 e 14,9 ± 8,0 mg/kg/dia no grupo controle (p = 0,001; 0,006 e 0,008, respectivamente). As doses médias de tacrolimus foram significativamente menores no grupo de idosos em comparação aos controles aos 3, 6 e 12 meses: 0,050 ± 0,031; 0,039 ± 0,025 e 0,034 ± 0,021 mg/kg/dia vs. 0,068 ± 0,038; 0,060 ± 0,034 e 0,053 ± 0,031 mg/kg/dia, respectivamente (p = 0,004; 0,0001 e 0,001). Apesar das doses mais baixas, os níveis mínimos de tacrolimus foram semelhantes entre os grupos em todos os momentos avaliados, exceto no sexto mês, quando os níveis foram significativamente mais elevados no grupo de idosos (7,3 ± 4,2 vs. 5,9 ± 1,8 ng/mL; p < 0,05).

### Eventos Adversos Pós-Transplante

No primeiro mês pós-transplante, as taxas de infecção fúngica ou bacteriana não diferiram significativamente entre os grupos (44% nos idosos e 47,3% nos controles; p > 0,05). Dentre essas complicações, as infecções do trato urinário foram as mais frequentemente observadas (51,2%). A TFG estimada ao final do primeiro mês não diferiu entre os pacientes que apresentaram ou não infecção do trato urinário durante esse período (39 [29–61] vs. 46 [26–60] mL/min/1,73 m^2^; p = 0,7).

A Tabela 3 descreve os principais eventos adversos observados após o primeiro mês do transplante. Verificou-se que a leucopenia foi mais prevalente no grupo de idosos em comparação ao grupo controle (56,5% vs. 35,6%; p = 0,017). A linfopenia persistiu ao final do primeiro ano em 27,9% do grupo de idosos e em 14,6% do grupo controle (p > 0,05). A prevalência de trombocitopenia, por outro lado, foi semelhante entre os grupos (19%).

**Tabela 3:** Complicações pós-transplante na coorte total, no grupo controle e no grupo de idosos

Variável	Total (n, %)	Controle (n, IC 95%)	Idosos (n, IC 95%)	p
Leucopenia	62 (42,1)	36 (35,6; 26,9–4,2)	26 (56,5; 43,7–69,3)	0,017
Trombocitopenia	28 (19)	19 (18,8; 11,7–25,9)	9 (19,5; 9,3–29,7)	> 0,05
Nefrotoxicidade	6 (4)	4 (3,8; 0,0–7,8)	2 (4,2; 0,0–9,9)	> 0,05
NODAT	18 (13,1)	13 (12,2; 6,2–18,2)	5 (16,1; 5,9–26,3)	> 0,05
CMV	49 (32,4)	28 (26,9; 18,7–35,1)	21 (44,7; 31,8–57,6)	0,034
Infecção bacteriana/fúngica	43 (28,6)	25 (24,0; 16,0–32,0)	18 (39,1; 26,4–51,8)	0,059
Neoplasia	3 (2)	1 (1,0; 0,0–2,9)	2 (4,2; 0,0–9,9)	> 0,05
Outras	07 (4,6)	5 (4,8; 1,0–8,6)	2 (4,2; 0,0–9,9)	> 0,05

Abreviaturas – NODAT: diabetes de início recente pós-transplante; CMV: citomegalovírus.

A frequência de NODAT foi de 16,1% no grupo de idosos e 12,2% no grupo controle (p > 0,05). A infecção por CMV foi mais comum no grupo de idosos (44,7% vs. 26,9%; p = 0,034), e observou-se uma tendência para maior ocorrência de infecções bacterianas e/ou fúngicas com necessidade de hospitalização nesse grupo (39,1% vs. 24%; p = 0,059).

### Desfechos Pós-Transplante

A Tabela 4 resume os desfechos pós-transplante. A rejeição aguda tratada ocorreu em 3,7% dos pacientes idosos e em 3,6% dos controles (p > 0,05). A nefrectomia do enxerto foi realizada em 10% dos pacientes em ambos os grupos. Não foi observada diferença entre os grupos na TFGe de 12 meses (57,7 ± 29,6 vs. 66,2 ± 29,1 mL/min/1,73 m^2^; p = 0,075). A Figura 1 apresenta a TFGe entre os pacientes vivos durante o acompanhamento de 12 meses.

**Tabela 4:** Desfechos sintetizando os resultados pós-transplante na coorte total, no grupo controle e no grupo de idosos

Parâmetros	Total (n, %)	Controle (n, IC 95%)	Idosos (n, IC 95%)	p
Rejeição Aguda	6 (3,7)	4 (3,6; 0,1–7,1)	2 (3,7; 0,0–9,1)	> 0,05
Nefrectomia de Enxerto	10 (6,1)	7 (6,4; 1,8–11,0)	3 (5,6; 0,0–11,7)	
Vascular	5 (3)	3 (2,7; 0,0–5,8)	2 (3,7; 0,0–9,1)	
Função não primária	2 (1,2)	2 (1,8; 0,0–4,4)	0 (0)	> 0,05
Fístula urinária	1 (0,6)	1 (0,9; 0,0–2,8)	0 (0)	
Choque hemorrágico	1 (0,6)	1 (0,9; 0,0–2,8)	0 (0)	
Indeterminado	1 (0,6)	0 (0)	1 (1,8; 0,0–5,3)	
Óbito	13 (8)	5 (4,6; 0,8–8,4)	8 (15,1; 5,8–24,4)	
Cardiovascular	5 (3)	1 (0,9; 0,0–2,8)	4 (7,5; 0,7–14,3)	
Infecção	4 (2,4)	2 (1,8; 0,0–4,4)	2 (3,7; 0,0–9,1)	**0,021**
Choque hemorrágico	3 (1,8)	2 (1,8; 0,0–4,4)	1 (1,8; 0,0–5,3)	
Indeterminado	1 (0,6)	0 (0)	1 (1,8; 0,0–5,3)	

**Figura 1:** TFG estimada nos 12 meses após o transplante.

Na coorte de idosos, cinco pacientes foram a óbito na primeira semana pós-transplante, em comparação a apenas dois casos de mortalidade precoce no grupo controle. Ao final do primeiro ano, o grupo de idosos apresentou um risco aumentado de óbito (sHR: 4,65; IC 95%: 1,22 – 17,7; p = 0,024) e exibiu menor sobrevida global em comparação com o grupo controle (86,4% vs 95,6%; p = 0,014). No entanto, a sobrevida do enxerto não diferiu significativamente entre os grupos (idosos: 86,8% vs. controle: 92,7%; p = 0,780) (Figura 2). Na análise multivariada, incluindo idade, diabetes, painel de reatividade de anticorpos > 20%, índice de massa corporal e doença cardiovascular prévia, nenhuma variável foi independentemente associada à mortalidade.

**Figura 2:** Análise de risco competitivo entre óbito do paciente e perda do enxerto.

## Discussão

Em nossa análise, constatamos que os RTR idosos apresentavam maior probabilidade de receber um regime imunossupressor de manutenção livre de corticosteroides e receberam doses significativamente mais baixas de micofenolato em comparação ao grupo mais jovem. Embora apresentassem níveis sanguíneos semelhantes de tacrolimus, foram observadas relações nível-dose mais elevadas desse medicamento nos idosos. Apesar de a sobrevida do enxerto censurada para óbito em um ano ter sido semelhante à dos controles, eles apresentaram uma taxa mais baixa de sobrevida global em um ano, impulsionada principalmente por óbitos ocorridos na primeira semana pós-transplante.

O protocolo de imunossupressão de nossa instituição não exclui explicitamente indivíduos idosos dos regimes de corticosteroides. No entanto, a prednisona foi utilizada com menor frequência em nossos pacientes idosos, provavelmentedevido à maior prevalência de diabetes e menor risco imunológico. Em contrapartida, o grupo controle apresentou maior incidência de DSA antes do transplante, em consonância com a observação de que pacientes mais jovens normalmente enfrentam um risco imunológico mais elevado^8^. Os inibidores da calcineurina podem permitir a não utilização ou a retirada precoce dos corticosteroides sem aumentar o risco de rejeição em pacientes com baixo risco imunológico. Dada a maior vulnerabilidade dos idosos ao NODAT, às doenças cardiovasculares e à osteoporose, essa abordagem mostra-se especialmente benéfica nessa população^9,10^. Tal estratégia pode aprimorar a sobrevida de longo prazo dos pacientes e contribuir para mitigar essas comorbidades^11^. De fato, apesar do risco basal mais elevado, nossos pacientes idosos apresentaram uma incidência de NODAT semelhante à dos controles.

Embora nosso protocolo não ajuste as doses de micofenolato com base na idade, doses mais baixas nos pacientes idosos provavelmente refletiram maiores taxas de leucopenia, infecção por CMV e aumento de infecções bacterianas/fúngicas que exigiram hospitalização depois do primeiro mês pós-transplante. Uma dose média mais baixa de micofenolato de mofetila em idosos um ano após o transplante já havia sido relatada anteriormente^12^, embora, no referido estudo, as taxas de infecção por CMV tenham sido semelhantes nos grupos de idosos e não idosos. É importante ressaltar que a redução das doses de micofenolato e do uso de esteroides em nossa coorte de idosos não esteve associada a um aumento significativo nas taxas de rejeição. Além disso, em um estudo anterior, a menor exposição a medicamentos imunossupressores foi associada a uma melhor sobrevida do enxerto em indivíduos com idade mais avançada, mesmo após a contabilização dos óbitos^13^. Por outro lado, uma maior exposição foi associada a um risco aumentado de óbito e perda do enxerto.

Após o terceiro mês do transplante, a coorte de idosos apresentou uma diferença pronunciada na relação nível-dose de TAC em comparação ao grupo controle. Jacobson et al.^14^ observaram uma relação nível-dose de TAC 68% menor nos idosos em comparação aos indivíduos mais jovens (129,8 vs. 77,1 ng/mL/mg/kg; p < 0,05). De forma semelhante, David-Neto et al.^15^ demonstraram que a dose média de TAC foi significativamente menor em indivíduos idosos (8,6 ± 4,8 mg vs. 12,1 ± 5,1 mg; p < 0,05) para alcançar níveis sanguíneos de TAC comparáveis aos de pacientes mais jovens.

Em nossa coorte, as taxas de rejeição aguda tratada foram comparáveis, apesar de o grupo de idosos apresentar maior número de incompatibilidades HLA, provavelmente devido às políticas de alocação que favorecem doadores com mais de 50 anos para receptores de idade mais avançada, uma prática comum entre os centros de transplante. Tullius et al.^6^ relataram uma taxa de rejeição de 10% em pacientes com idade superior a 65 anos, o que representa metade da taxa observada em indivíduos entre 20 e 30 anos. A baixa taxa de rejeição (3,6% em ambos os grupos), comparada aos dados publicados, pode refletir a alta exposição a imunossupressores, o menor número de receptores e doadores de alto risco e o tamanho amostral limitado do estudo.

A prevalência de infecção por CMV foi significativamente maior no grupo de idosos em comparação ao grupo controle (44,7% vs. 26,9%). Pesquisas indicam que a infecção por CMV ocorre em mais de 60% dos pacientes transplantados de alto risco^16^. O micofenolato demonstrou aumentar o risco de infecção por CMV na população idosa^13^, enquanto os inibidores de mTOR parecem oferecer um efeito protetor contra o CMV^17^. Em adultos com idade mais avançada, os inibidores de mTOR demonstram farmacocinética estável durante os primeiros seis meses após o transplante, atingindo níveis estáveis do medicamento por volta do sétimo dia, o que indica que, em geral, não são necessários ajustes significativos na dose^15^.

Dados internacionais não têm demonstrado de forma consistente diferenças significativas nas taxas globais de sobrevida de pacientes e enxertos em 1 e 5 anos após o transplante^12^. No entanto, a menor sobrevida em nosso grupo de idosos é consistente com os achados de um estudo brasileiro anterior que relatou diferenças semelhantes na sobrevida entre receptores idosos e mais jovens^18^. Essa disparidade tem sido atribuída ao aumento da incidência de comorbidades, como diabetes, que é um fator de risco bem conhecido para mortalidade precoce após transplante renal^19^. Além disso, foi relatado que o risco relativo de óbito por infecção é fortemente influenciado pela idade^4^. Em nosso estudo, a maioria dos óbitos em idosos ocorreu na primeira semana pós-transplante, principalmente por causas cardiovasculares e infecções. Apesar da incidência basal mais elevada de diabetes e doença cardiovascular, nenhum fator previu independentemente a mortalidade, ressaltando a complexa interação de variáveis—incluindo potencialmente fatores não mensuráveis, como fragilidade—nos desfechos de 12 meses pós-transplante em idosos. É importante ressaltar que dados prévios mostraram que, embora RTR idosos apresentem um risco aumentado de mortalidade precoce pós-transplante, eles experimentam uma melhora progressiva e substancial na sobrevida em longo prazo e devem apresentar taxas de sobrevida superiores quando comparados àqueles que permanecem em diálise^20^.

Embora limitado por seu desenho retrospectivo e dados incompletos sobre adesão, estado imunológico e fragilidade geriátrica, este estudo se beneficia de um protocolo imunossupressor padronizado e perfis consistentes de doadores.

Nossos achados sugerem que RTR idosos podem se beneficiar de uma exposição reduzida a agentes imunossupressores, em consonância com a literatura existente. A seleção cuidadosa dos candidatos e os cuidados perioperatórios personalizados permanecem essenciais para a otimização dos desfechos nessa população crescente de pacientes.

## Data Availability

O conjunto completo de dados que sustenta os achados deste estudo está disponível mediante solicitação à autora correspondente, Aline Cunha Lima Alcântara. O conjunto de dados não está disponível publicamente devido à presença de informações clínicas sensíveis que poderiam comprometer a privacidade dos participantes.
